# Metformin: an old but still the best treatment for type 2 diabetes

**DOI:** 10.1186/1758-5996-5-6

**Published:** 2013-02-15

**Authors:** Lilian Beatriz Aguayo Rojas, Marilia Brito Gomes

**Affiliations:** 1 Department of Medicine, Diabetes Unit, State University of Rio de Janeiro, Av 28 setembro 77, Rio de Janeiro CEP20555-030, Brazil

**Keywords:** Metformin, Diabetes mellitus, Insulin, Resistance

## Abstract

The management of T2DM requires aggressive treatment to achieve glycemic and cardiovascular risk factor goals. In this setting, metformin, an old and widely accepted first line agent, stands out not only for its antihyperglycemic properties but also for its effects beyond glycemic control such as improvements in endothelial dysfunction, hemostasis and oxidative stress, insulin resistance, lipid profiles, and fat redistribution. These properties may have contributed to the decrease of adverse cardiovascular outcomes otherwise not attributable to metformin’s mere antihyperglycemic effects. Several other classes of oral antidiabetic agents have been recently launched, introducing the need to evaluate the role of metformin as initial therapy and in combination with these newer drugs. There is increasing evidence from *in vivo* and *in vitro* studies supporting its anti-proliferative role in cancer and possibly a neuroprotective effect. Metformin’s negligible risk of hypoglycemia in monotherapy and few drug interactions of clinical relevance give this drug a high safety profile. The tolerability of metformin may be improved by using an appropiate dose titration, starting with low doses, so that side-effects can be minimized or by switching to an extended release form. We reviewed the role of metformin in the treatment of patients with type 2 diabetes and describe the additional benefits beyond its glycemic effect. We also discuss its potential role for a variety of insulin resistant and pre-diabetic states, obesity, metabolic abnormalities associated with HIV disease, gestational diabetes, cancer, and neuroprotection.

## Introduction

The discovery of metformin began with the synthesis of galegine-like compounds derived from *Gallega officinalis*, a plant traditionally employed in Europe as a drug for diabetes treatment for centuries [1]. In 1950, Stern *et al.* discovered the clinical usefulness of metformin while working in Paris. They observed that the dose–response of metformin was related to its glucose lowering capacity and that metformin toxicity also displayed a wide security margin [1].

Metformin acts primarily at the liver by reducing glucose output and, secondarily, by augmenting glucose uptake in the peripheral tissues, chiefly muscle. These effects are mediated by the activation of an upstream kinase, liver kinase B1 (LKB-1), which in turn regulates the downstream kinase adenosine monophosphatase protein kinase (AMPK). AMPK phosphorylates a transcriptional co-activator, transducer of regulated CREB protein 2 (TORC2), resulting in its inactivation which consequently downregulates transcriptional events that promote synthesis of gluconeogenic enzymes [2]. Inhibition of mitochondrial respiration has also been proposed to contribute to the reduction of gluconeogenesis since it reduces the energy supply required for this process [3].

Metformin’s efficacy, security profile, benefic cardiovascular and metabolic effects, and its capacity to be associated with other antidiabetic agents makes this drug the first glucose lowering agent of choice when treating patients with type 2 diabetes mellitus (TDM2).

### Metformin and pre-diabetes

In 2000, an estimated 171 million people in the world had diabetes, and the numbers are projected to double by 2030. Interventions to prevent type 2 diabetes, therefore, have an important role in future health policies. Developing countries are expected to shoulder the majority of the burden of diabetes [4]. One of the main contributing factors to this burden is the Western lifestyle which promotes obesity and sedentarism [5].

Impaired glucose tolerance (IGT) and impaired fasting glucose (IFG) statuses are associated with increased and varying risk of developing type 2 diabetes mellitus. IGT has been associated with an increased risk of cardiovascular events and may determine an increased mortality risk. The association of IFG with cardiovascular events, however, has not been well established [6].

When lifestyle interventions fail or are not feasible, pharmacological therapy may be an important resource to prevent type 2 diabetes. Several different drug classes have been studied for this purpose.

In their systematic review, Gillies *et al.* found that lifestyle and pharmacological interventions reduced the rate of progression to type 2 diabetes in people with IGT and that these interventions seem to be as effective as pharmacological treatment. Although compliance was high, treatment effect was not sustained after treatment was stopped. According to the results of their meta-analysis, lifestyle interventions may be more important in those with higher mean baseline body mass index BMI [5].

The best evidence for a potential role for metformin in the prevention of type 2 diabetes comes from The Diabetes Prevention Program (DPP) trial. Lifestyle intervention and metformin reduced diabetes incidence by 58% and 31%, respectively, when compared with placebo [7].

At the end of the DPP study, patients were observed for a one to two week wash out period. Diabetes incidence increased from 25.2 to 30.6% in the metformin group and from 33.4 to 36.7% in the placebo group. Even after including the wash out period in the overall analysis, metformin still significantly decreased diabetes incidence (risk ratio 0.75, p = 0.005) compared with placebo [8]. These data suggest that, at least in the short-term, metformin may help delay the onset of diabetes. The benefits of metformin were primarily observed in patients <60 years old (RR 0.66) and in patients with a BMI greater than 35 kg/m^2^ (RR 0.47) [7] (Table 1).

**Table 1:** Effectiveness of metformin in diabetes prevention of patients with impaired glucose tolerance

**Study**	**Randomized**	**Country**	**N**	**Duration years**	**Mean change in risk MET (%)**	**Mean change in risk LSM (%)**
DPP [7]	yes	USA	3234	3	−31%	−58%
IDPP [9]	yes	India	522	3	−26.4%	−28.2%
Yang *et al.*[10]	yes	China	321	2.5	−77%	-
DPPOS [11]	yes	USA	2766	5.7	−18%	−34%

DPP: Diabetes Prevention Program, DPP: Indian Diabetes Prevention Program, DPPOS: Diabetes Prevention Outcome Study, MET: Metformin, LSM: Lifestyle modification.

Metformin significantly reduced the risk of developing diabetes in an Indian population of subjects with IGT. The relative risk reduction was 28.5% with lifestyle modification (p = 0.018), 26.4% with metformin (p = 0.029), and 28.2% with lifestyle modification plus metformin (p = 0.022), as compared with the control group [9] (Table 1).

In a Chinese study, subjects with IGT randomly assigned to receive either low-dose metformin (750 mg/day) or acarbose (150 mg/day) in addition to lifestyle intervention were compared to a control group that only received life style intervention. Treatment with metformin or acarbose produced large, significant, and similar risk reductions for new onset of T2DM of 77% and 88%, respectively; these reductions were larger than that of lifestyle intervention alone [10].

The persistence of the long-term effects obtained through DPP interventions were evaluated at an additional follow-up after a median of 5.7 years. Individuals were divided in 3 groups: lifestyle, metformin, and placebo. Diabetes incidence rates were similar between treatment groups: 5.9 per 100 person-years (5.1–6.8) for lifestyle, 4.9 (4.2–5.7) for metformin, and 5.6 (4.8–6.5) for placebo. Diabetes incidence 10 years since DPP randomization was reduced by 34% and 18% in the lifestyle and metformin group, respectively [11] (Table 1).

The prevalence of pre-diabetes as well as the progression rate to diabetes may differ between different populations, making the application of results from certain studies of different ethnical groups inappropriate. IGT is highly prevalent in native Asian Indians. This population has several unique features such as a young age of diabetes onset and lower BMI along with high rates of insulin resistance and lower thresholds for diabetic risk factors [12]. Chinese individuals have a lower prevalence of diabetes and are less insulin resistant than Indians, so the results of the Chinese study may not be applicable to Asian Indian individuals [13].

In a meta-analysis of randomized controlled trials, Salpeter *et al.* reported a reduction of 40% in the incidence of new-onset diabetes with an absolute risk reduction of 6% (95% CI, 4–8) during a mean trial duration of 1.8 years [14].

Lily and Godwin reported a decreased rate of conversion from pre-diabetes to diabetes in individuals with IGT or IFG in their systematic review and meta-analysis of randomized controlled trials. This effect was seen at both a higher metformin dosage (850 mg twice daily) and lower metformin dosage (250 mg twice or 3 times daily) in people of varied ethnicity [15].

### Metformin in the management of adult diabetic patients

Current guidelines from the American Diabetes Association/European Association for the Study of Diabetes (ADA/EASD) and the American Association of Clinical Endocrinologists/American College of Endocrinology (AACE/ACE) recommend early initiation of metformin as a first-line drug for monotherapy and combination therapy for patients with T2DM. This recommendation is based primarily on metformin’s glucose-lowering effects, relatively low cost, and generally low level of side effects, including the absence of weight gain [16,17].

Metformin’s first-line position was strengthened by the United Kingdom Prospective Diabetes Study (UKPDS) observation that the metformin-treated group had risk reductions of 32% (p = 0.002) for any diabetes-related endpoint, 42% for diabetes-related death (p = 0.017), and 36% for all-cause mortality (p = 0.011) compared with the control group. The UKPDS demonstrated that metformin is as effective as sulfonylurea in controlling blood glucose levels of obese patients with type 2 diabetes mellitus [18]. Metformin has been also been shown to be effective in normal weight patients [19].

### Metformin in combination therapy

Although monotherapy with an oral hypoglycemic agent is often initially effective, glycemic control deteriorates in most patients which requires the addition of a second agent. Currently, marketed oral therapies are associated with high secondary failure rates [20]. Combinations of metformin and insulin secretagogue can reduce HbA1c between 1.5% to 2.2% in patients sub-optimally controlled by diet and exercise [21].

The optimal second-line drug when metformin monotherapy fails is not clear. All noninsulin antidiabetic drugs when added to maximal metformin therapy are associated with similar HbA1c reduction but with varying degrees of weight gain and hypoglycemia risk. A meta-analysis of 27 randomized trials showed that thiazolidinediones, sulfonylureas, and glinides were associated with weight gain; glucagon-like peptide-1 analogs, glucosidase inhibitors, and dipeptidyl peptidase-4 inhibitors were associated with weight loss or no weight change. Sulfonylureas and glinides were associated with higher rates of hypoglycemia than with placebo. When combined with metformin, sulfonylureas and alpha-glucosidase inhibitors show a similar efficacy on HbA1c [22].

### Metformin and sulfonylureas

The combination of metformin and sulfonylurea (SU) is one of the most commonly used and can attain a greater reduction in HbA1c (0.8–1.5%) than either drug alone [23,24]. The glimepiride/metformin combination results in a lower HbA1c concentration and fewer hypoglycemic events when compared to the glibenclamide/metformin combination [25]. The use of metformin was associated with reduced all-cause mortality and reduced cardiovascular mortality. Metformin and sulfonylurea combination therapy was also associated with reduced all-cause mortality [26].

Epidemiological investigations suggest that patients on SUs have a higher cardiovascular disease event rate than those on metformin. Patients who started SUs first and added metformin also had higher rates of cardiovascular disease events compared with those who started metformin first and added SUs. These investigations are potentially affected by unmeasured confounding variables [27].

### Metformin and insulin

Metformin as added to insulin-based regimens has been shown to improve glycemic control, limit changes in body weight, reduce hypoglycemia incidence, and to reduce insulin requirements (sparing effect), allowing a 15–25% reduction in total insulin dosage [28,29].

The addition of metformin to insulin therapy in type 1 diabetes is also associated with reductions in insulin-dose requirement and HbA1c levels [30,31].

### Metformin and thiazolinediones

The addition of rosiglitazone to metformin in a 24-week randomized, double-blind, parallel-group study significantly decreased HbA1c concentration and improved insulin sensitivity and HOMA ß cell function [32]. However, in spite of preventing diabetes incidence, the natural course of declining insulin resistance may not be modified by a low dose of the metformin-rosiglitazone combination [33].

The ADOPT study (A Diabetes Outcome Progression Trial) assessed the efficacy of rosiglitazone, as compared to metformin or glibenclamide, in maintaining long-term glycemic control in patients with recently diagnosed type 2 diabetes. Rosiglitazone was associated with more weight gain, edema, and greater durability of glycemic control; metformin was associated with a higher incidence of gastrointestinal events and glibenclamide with a higher risk of hypoglycaemia. [34].

### Metformin and glifozins

Dapagliflozin, a highly selective inhibitor of SGLT2, has demonstrated efficacy, alone or in combination with metformin, in reducing hyperglycemia in patients with type 2 diabetes [35,36]. Studies are in development for assessing the safety and efficacy of this combination.

### Metformin and α glicosidase inhibitor

Acarbose reduces the bioavailability of metformin [37]. However, it has been reported that the association of acarbose to metformin in sub-optimally controlled patients reduced HbA1c by about 0.8-1.0% [38].

### Metformin and incretin-based therapies

DDPIV prolongs the duration of active glucagon-like peptide 1 (GLP-1) by inhibiting DPPIV peptidase, an enzyme which cleaves the active form of the peptide. This action results in an improvement of insulin secretion as a physiological response to feeding. The mechanism of DPPIV inhibitors is complementary to that of metformin which improves insulin sensitivity and reduces hepatic glucose production, making this combination very useful for achieving adequate glycemic control [39]. Metformin has also been found to increase plasma GLP-1 levels, probably by either direct inhibition of DPPIV or by increased secretion, leading to reduced food intake and weight loss [40].

Saxagliptin added to metformin led to clinically and statistically significant reductions in HbA1c from baseline versus metformin/placebo in a 24-week randomized, double-blind, placebo-controlled trial. Saxagliptin at doses of 2.5, 5, and 10 mg plus metformin decreased A1 by 0.59%, 0.69%, and 0.58%, respectively, in comparison to an increase in the metformin plus placebo group (+0.13%); p < 0.0001 for all comparisons [41].

A meta-analysis of 21 studies examined incretin-based therapy as an add-on to metformin in patients with T2DM for 16–30 weeks; 7 studies used a short-acting GLP-1 receptor agonist (exenatide BID), 7 used longer acting GLP-1 receptor agonists (liraglutide or exenatide LAR), and 14 examined DPP-IV inhibitors. Long-acting GLP-1 receptor agonists reduced HbA1c and fasting glucose levels to a greater extent than the other therapies [42].

### Metformin and pregnancy

Metformin is known to cross the placenta and concerns regarding potential adverse effects on both the mother and the fetus have limited its use in pregnancy [43]. The use of metformin during pregnancy is still a matter of controversy.

Two meta-analyses of observational studies, one of women using metformin and/or sulfonylureas and one of women using metformin alone during the first trimester, did not show an increase in congenital malformations or neonatal deaths [44,45].

The Metformin in Gestational Diabetes (MiG) trial, found no significant difference in the composite fetal outcome (composite of neonatal hypoglycemia, respiratory distress, need for phototherapy, birth trauma, 5-minute Apgar score <7, or prematurity) between metformin and insulin. Women assigned to metformin had more preterm births and less weight gain compared to those in the insulin group [46]. Another randomized trial also found similar results [47].

Results of the MiG TOFU reported that infants of diabetic mothers exposed to metformin in utero and examined at 2 years of age may present a reduction in insulin resistance, probably related to an increase in subcutaneous fat [48].

Longer follow-up studies will be required to determine metformin’s impact on the development of obesity and metabolic syndrome in offspring.

### Metformin use in childhood and adolescence

Type 2 diabetes mellitus has dramatically increased in children and adolescents worldwide to the extent that has been labeled an epidemic [49]. Before 1990, it was a rare condition in the pediatric population; by 1999, the incidence varied from 8% to 45%, depending on geographic location, and was disproportionally represented among minority groups [50]. There are few studies of metformin use in the pediatric population. Most of them are of short duration and heterogeneous designs.

The beneficial role of metformin in young patients with type 2 diabetes has been demonstrated in a randomized, controlled trial which showed a significant decrease in fasting blood glucose, HbA1c, weight, and total cholesterol. The most frequently reported adverse events were abdominal pain, diarrhea, nausea/vomiting, and headaches. There were no cases of clinical hypoglycemia, lactic acidosis, or clinically significant changes in physical examinations [51]. When compared to glimepiride (1–8 mg once daily), metformin (500–1000 mg twice daily) lowered HbA1c to <7%, similar to glimepiride, but was associated with significantly less weight gain. A total of 42.4% and 48.1% of subjects in the glimepiride and metformin groups, respectively, in the intent-to-treat population achieved A1C levels of <7.0% at week 24 [52].

There is some evidence that suggests improvement in metabolic control of poorly controlled adolescents with type 1 diabetes when metformin is added to insulin therapy. Metformin has been shown to reduce insulin dose requirement (5.7–10.1 U/day), HbA1c (0.6–0.9%), weight (1.7–6.0 kg), and total cholesterol (0.3–0.41 mmol/l) [30]. A previous review showed similar results in HbA1 reduction and insulin requirement, however no improvements in insulin sensitivity, body composition, or serum lipids were documented [31].

### Metformin indications for management of obesity, insulin resistance, and non-alcoholic fatty liver in children and adolescents

Insulin resistance in obese children and adolescents should be appropriately and aggressively addressed once it is linked to known cardiovascular risks such as IGT, T2DM, dyslipidemia, and hypertension [53,54]. Non-alcoholic fatty (NAFLD) disease, a frequent cause of chronic liver disease in obese adults, is also associated with a higher risk of developing diabetes and of progression to fibrosis and cirrhosis [55] with an increased relative risk of cardiovascular events or death [56]. The true prevalence of NAFLD in children is underestimated. The prevalence of steatosis in obese children was estimated to be 38% in a large retrospective autopsy study [57].

Currently, the best supported therapy for NAFLD is gradual weight loss through exercise and nutritional support [58]. Metformin is associated with short-term weight loss, improvement of insulin sensitivity, and decreased visceral fat [59]. A reduction in ALT, GGT, and fatty liver incidence and severity has also been described with metformin use [60].

Metformin has been used increasingly in obese children with hyperinsulinemia although there are no strong evidence-based studies supporting its use for this clinical condition. A moderate improvement in body muscular index (BMI) and insulin sensitivity has been reported with the use of metformin [61,62]. Heart rate recovery (HRR) may also improve due to improved parasympathetic tone, paralleling improvements in BMI, insulin levels, and insulin sensitivity [61]. HRR has been considered a predictor of mortality and cardiovascular disease in otherwise healthy subjects [63]. A poor HRR has also been linked to insulin resistance [64] and to a higher risk for developing T2DM [65].

Metformin may not be as effective as behavioral interventions in reducing BMI and when compared with drugs that are licensed for obesity, its effects are moderate [66].

### Effects of metformin on vascular protection

#### Effects on cardiovascular mortality

Diabetic patients are at high risk of cardiovascular events, particularly of coronary heart disease by about 3-fold [67,68]. It has been stated that type 2 diabetic patients without a previous history of myocardial infarction have the same risk of coronary artery disease (CAD) as non-diabetic subjects with a history of myocardial infarction [69]. This has led the National Cholesterol Education Program to consider diabetes as a coronary heart disease risk equivalent [70]. Although there is no doubt that there is an increased risk of CAD events in diabetic patients, there is still some uncertainty as to whether the cardiovascular risk conferred by diabetes is truly equivalent to that of a previous myocardial infarction [71].

In 1980, Scambato *et al.* reported that, in a 3-year observational study of 310 patients with ischaemic cardiomyopathy, patients treated with metformin had reduced rates of re-infarction, occurrence of angina pectoris, acute coronary events other than acute myocardial infarction, and death in patients [72]. The largest effect was seen in re-infarction rates; a post hoc analysis showed that this effect was significant (p = 0.003). After this study, the UKPDS, the largest randomized clinical trial in the newly-diagnosed type 2 diabetic population largely free of prior major vascular events, randomly assigned treatment with metformin to a subgroup of overweight individuals (>120% of ideal body weight). In 1990, another subgroup of patients (n = 537), who were receiving the maximum allowed dosage of sulfonylurea, were randomized either to continue sulfonylurea therapy or to allow an early addition of metformin [18].

Metformin provided greater protection against the development of macrovascular complications than would be expected from its effects upon glycemic control alone. It had statistically significant reductions in the risk of all-cause mortality, diabetes-related mortality (p = 0.017), and any end-point related to diabetes (p = 0.002), but not in myocardial infarction (p = 0.052) [18]. The UKPDS pos-trial reported significant and persistent risk reductions for any diabetes-related end point (21%, p = 0.01), myocardial infarction (33%, p = 0.005), and death from any cause (27%, p = 0.002) [73].

Following UKPDS, other studies have reported significant improvement of all-cause mortality and cardiovascular mortality (Table 2). A retrospective analysis of patients databases in Saskatchewan, Canada reported significant reductions for all-cause mortality and cardiovascular mortality of 40% and 36%, respectively [26]. The PRESTO trial showed significant reductions of any clinical event (28%), myocardial infarction (69%), and all-cause mortality (61%) [74]. The HOME trial reported a decreased risk of developing macrovascular disease [75]. In non-diabetic subjects with normal coronary arteriography but also with two consecutive positive (ST depression > 1 mm) exercise tolerance test, an 8-week period on metformin improved maximal ST-segment depression, Duke score, and chest pain incidence compared with placebo [76]. A recent meta-analysis suggested that the cardiovascular effects of metformin could be smaller than had been hypothesized on the basis of the UKPDS; however, its results must be interpreted with caution given the low number of randomized controlled trials included [77].

**Table 2:** Metformin effects on vasculoprotection

**Study**	**Design**	**Duration**	**Key findings**
UKPDS 33 [18]	Prospective	10 yr	Significant reduction in all-cause mortality, diabetes related mortality, and any end-point related to diabetes.
Sgambato *et al.*[72]	Retrospective	3 yr	Trend towards reduction in angina symptoms (p = 0.051). Significant lower re-infarction rates.
Johnson *et al.*[24]	Retrospective	9 yr	Reduction of all-cause mortality and of cardiovascular mortality
Kao *et al.*[74]	Prospective	2 yr	Significant risk reduction for any clinical event, myocardial infarction and all-cause mortality
Jadhav *et al.*[76]	Prospective	8 weeks	Improved maximal ST depression, Duke score, and chest pain incidence
Kooy *et al.*[75]	Prospective	4, 3 yr	Reduction of the risk of developing macrovascular disease

### Metformin and heart failure

The risk of developing cardiac heart failure (CHF) in diabetic individuals nearly doubles as the population ages [77]. DM and hyperglycemia are strongly implicated as a cause for the progression from asymptomatic left ventricular dysfunction to symptomatic HF, increased hospitalizations for HF, and an overall increased mortality risk in patients with chronic HF [78]. Despite all its benefits, metformin is contraindicated in patients with heart failure due to the potential risk of developing lactic acidosis, a rare but potentially fatal metabolic condition resulting from severe tissue hypoperfusion [79]. The US Food and Drug Administration removed the heart failure contraindication from the packaging of metformin although a strong warning for the cautious use of metformin in this population still exists [80].

Several retrospective studies in patients with CHF and diabetes reported lower risk of death from any cause [81-83], lower hospital readmissions for CHF [81], and hospitalizations for any cause [81,82]. A recent review concluded that CHF could not be considered an absolute contraindication for metformin use and also suggest its protective effect in reducing the incidence of CHF and mortality in T2DM [83]. This protective effect may due to AMPK activation and decrease in cardiac fibrosis [83].

In a prospective 4-year study, 393 metformin-treated patients with elevated serum creatinine between 1.5–2.5 mg/dL and coronary artery disease, CHF, or chronic obstructive pulmonary disease (COPD) were randomized into two groups. One group continued metformin therapy while the other was instructed to discontinue metformin. Patients with CHF had either New York Heart Association (NYHA) Class III or Class IV CHF and were receiving diuretic and vasodilatation drugs. There were no differences between groups in all-cause mortality, cardiovascular mortality, rate of myocardial infarction, or rate of cardiovascular events [84].

Patients with DM and advanced, systolic HF (n = 401) were divided into 2 groups based on the presence or absence of metformin therapy. The cohort had a mean age of 56 ± 11 years and left ventricular ejection fraction (LVEF) of 24 ± 7% with 42% and 45% being NYHA III and NYHA IV, respectively. Twenty-five percent (n = 99) were treated with metformin therapy. Metformin-treated patients had a higher BMI, lower creatinine, and were less often on insulin. One-year survival in metformin-treated and non-metformin-treated patients was 91% and 76%, respectively (p = 0.007). After a multivariate adjustment for demographics, cardiac function, renal function, and HF medications, metformin therapy was associated with a non-significant trend of improved survival [85].

Many different mechanisms, beyond glycemic control, have been implicated in vascular protection induced by metformin such as improvements in the inflammatory pathway [86], coagulation [87], oxidative stress and glycation [88-92], endothelial dysfunction [88-90], haemostasis [88,91-93], insulin resistance improvement [94], lipid profiles [95,96], and fat redistribution [97,98]. Some of these mechanisms are described below.

### Beyond glycemic control

The UKPDS recruited patients with newly diagnosed type 2 diabetes and demonstrated that tight glycemic control has beneficial effects on microvascular end points. However, it failed to show improvements in macrovascular outcomes. The improved cardiovascular disease (CVD) risk in overweight diabetic patients treated with metformin was attributed to its effects extending beyond glycemic control [18].

### Effects on the inflammatory pathway

The benefits of metformin on macrovascular complications of diabetes, separate from its conventional hypoglycemic effects, may be partially explained by actions beyond glycemic control, particularly by actions associated with inflammatory and atherothrombotic processes [86]. Metformin can act as an inhibitor of pro-inflammatory responses through direct inhibition of NF-kB by blocking the PI3K–Akt pathway. This effect may partially explain the apparent clinical reduction of cardiovascular events not fully attributable to metformin’s anti-hyperglycemic action [86].

Some studies also point to a modest effect on inflammatory markers in subjects with IGT in T2DM [87] while others have found no effect at all [88].

### Effects on oxidative stress

Oxidative stress is believed to contribute to a wide range of clinical conditions such as inflammation, ischaemia-reperfusion injury, diabetes, atherosclerosis, neurodegeneration, and tumor formation [99].

Metformin has antioxidant properties which are not fully characterized. It reduces reactive oxygen species (ROS) by inhibiting mitochondrial respiration [100] and decreases advanced glycosylation end product (AGE) indirectly through reduction of hyperglycemia and directly through an insulin-dependent mechanism [101].

There is some evidence that metformin also has a beneficial effect on some components of the antioxidant defense system. It can upregulate uncoupled proteins 2 (UCP2) in adipose tissue [102] and can also cause an increase in reduced glutathione [100].

Metformin has been proposed to cause a mild and transient inhibition of mitochondrial complex I which decreases ATP levels and activates AMPK-dependent catabolic pathways [100], increasing lipolysis and ß-oxidation in white adipose tissue [102] and reducing neoglucogenesis [2]. The resultant reduction in triglycerides and glucose levels could decrease metylglyoxal (MG) production through lipoxidation and glycoxidation, respectively [99,101].

Recently a study described a putative mechanism relating metformin action and inhibition of oxidative stress, inflammatory, and proapoptotic markers [103]. In this study, treatment of bovine capillary endothelial cells incubated in hyperglycemic medium with metformin was able to decrease the activity of NF-kB and others intracellular proteins related to cellular metabolic memory. The authors suggested that this action could be mediated by histone deacetylase sirtuin 1 (SIRT-1), a multifunctional protein involved in many intracellular pathways related to metabolism, stress response, cell cycle, and aging [103].

### Effects on endothelial function

Type 2 diabetes is associated with a progressive and generalized impairment of endothelial function that affects the regulation of vasomotor tone, leucocyte adhesion, hemostasis, and fibrinolysis. These effects are probably direct and not related to decreases in hyperglycemia [88].

Contradictory effects of metformin on endothelial function have been described, however [89,90]. Mather *et al.* reported that metformin has no effect on endothelium dependent blood flow but has a significant effect on endothelium independent blood flow and insulin resistance reduction [89]. Conversely, Vitale *et al.* found significant improvement of endothelium dependent flow without a significant effect on endothelium independent response [90]. Further studies are necessary to establish the effect of metformin on endothelial function.

### Effects on body weight

Metformin may have a neutral effect on body weight of patients with T2DM when compared to diet [18] or may limit or decrease the weight gain experienced with sulfonylureas [18], TDZ [104], insulin [29,75], HAART [97], and antipsychotics drugs [94].

Modest weight loss with metformin has been observed in subjects with IGT [15,18]. However, a meta-analysis of overweight and obese non-diabetic subjects, found no significant weight loss as either a primary or as secondary outcome [105].

The mechanisms by which metformin contributes to weight loss may be explained through the reduction in gastrointestinal absorption of carbohydrates and insulin resistance [95], reduction of leptin [95] and ghrelin levels after glucose overload [96], and by induction of a lipolitic and anoretic effect by acting on glucagon–like peptide 1 [40].

### Effects on lipid profile

Metformin is associated with improvements in lipoprotein metabolism, including decreases in LDL-C [95], fasting and postprandial TGs, and free fatty acids [106].

### Effects on blood pressure

The hypertension associated with diabetes has an unclear pathogenesis that may involve insulin resistance. Insulin resistance is related to hypertension in both diabetic and non-diabetic individuals and may contribute to hypertension by increasing sympathetic activity, peripheral vascular resistance, renal sodium retention [107], and vascular smooth muscle tone and proliferation [108,109].

Data of the effects of metformin on BP are variable, with neutral effects or small decreases in SBP and DBP [110]. In the BIGPRO1 trial carried out in upper-body obese non-diabetic subjects with no cardiovascular diseases or contraindications to metformin, blood pressure decreased significantly more in the IFG/IGT subgroup treated with metformin compared to the placebo group (p < 0.03) [111].

### Effects on thyroid function

Metformin decreases serum levels of thyrotropin (TSH) to subnormal levels in hypothyroid patients that use levothyroxin (LT4) on a regular basis [112]. A significant decrease in TSH (P < 0.001) without reciprocal changes in any thyroid function parameter in diabetic patients had also been reported but only in hypothyroid subjects, not in euthyroid ones [113].

The mechanism of the drop in TSH is unclear at this time. Some of the proposed explanations for this effect are enhanced inhibitory modulation of thyroid hormones on central TSH secretion, improved thyroid reserve in patients with hypothyroidism [113], changes in the affinity or the number of thyroid hormone receptors, increased dopaminergic tone, or induced constituent activation of the TSH receptor [112].

### Metformin and HIV lypodystrofy

Antiretroviral therapy has been associated with an increased prevalence of type 2 diabetes mellitus and insulin resistance among HIV-infected patients [114]. Lipodystrophy, characterized by morphological (peripheral lipoatrophy, localized fat accumulation) and metabolic changes (hyperlipidemia, insulin resistance and hyperglycemia), is highly prevalent in patients on highly active antiretroviral therapy (HAART), occurring in 40% to 80% of patients [115].

Nucleoside reverse transcriptase inhibitors (NRTIs), particularly thymidine analogues (zidovudine and stavudine), have been associated with morphological changes, particularly extremity fat loss [116], while protease inhibitors (PIs) have been associated with biochemical derangements of glucose and lipids as well as with localized accumulation of fat [117].

Lifestyle modifications such as diet and exercise and switching antiretroviral therapies seems to be of limited value in reducing visceral abdominal fat (VAT). Metformin has been shown to reduce VAT [97,98] but at the expense of accelerating peripheral fat loss [118]. Favorable effects on insulin levels [98], insulin sensitivity [119], weight [97], flow-mediated vasodilation [119], and lipid profiles [98,119] have also been described.

### Effects on hemostasis

Therapeutic doses of metformin in type 2 diabetic patients lower circulating levels of several coagulation factors such as plasminogen activator inhibitor (PAI-1), von Williebrand Factor (vWF), tissue type plasminogen activator [88], factor VII [91]. It has also direct effects on fibrin structure and function by decreasing factor XIII activity and changing fibrin structure [92].

Furthermore, plasma levels of PAI-1 and vWF, which are secreted mainly by the impaired endothelium, have been shown to decrease with metformin therapy in non-diabetic subjects [93].

### Metformin and neuroprotection

Alzheimer’s disease (AD), one of the most common neurodegenerative diseases, has been termed type 3 diabetes. It is a brain specific form of diabetes characterized by impaired insulin actions and neuronal insulin resistance [120] that leads to excessive generation and accumulation of amyloid oligomers, a key factor in the development of AD [121].

The mechanisms of cerebral metabolism are still unclear. A network of different factors is most likely responsible for its maintenance. The activated protein kinase (AMPK) forms a molecular hub for cellular metabolic control [122]. Recent studies of neuronal models are pointing to possible AMPK roles beyond energy sensing with some reporting protective effects [123] while others report detrimental effects, particularly under extreme energy depletion [124].

AMPK is activated in the brain by metabolic stresses that inhibit ATP production such as ischemia, hypoxia, glucose deprivation, metabolic inhibitors (metformin), as well as catabolic and ATP consuming processes [122].

The human brain is characterized by an elevated oxidative metabolism and low antioxidants enzymes, which increases the brain’s vulnerability to oxidative stress [125]. Oxidative stress has been implicated in a variety of neurological diseases, including Alzheimer’s disease, Parkinson’s disease, and amyotrophic lateral sclerosis disease [126]. Mitochondrial dysfunction has a pivotal role in oxidative stress. In this setting, the permeability transition pore (PTP) acts as a regulator of the apoptotic cascade under stress conditions, triggering the release of apoptotic proteins and subsequent cell death [127]. It was reported that metformin prevents PTP opening and subsequent cell death in various endothelial cell types exposed to high glucose levels [128]. Metformin could interrupt the apoptotic cascade in a model of ectoposide-induced cell death by inhibiting PTP opening and blocking the release of cytochrome-c. These events together with other factors from the mitochondrial intermembrane space are critical processes in the apoptotic cascade [125].

Insulin has been shown to regulate a wide range of processes in the central nervous system such as food intake, energy homeostasis, reproduction, sympathetic activity, learning and memory [129], as well as neuronal proliferation, apoptosis, and synaptic transmission [130].

With regard to ß amyloid, a report has shown that metformin increases ß amyloid in cells through an AMPK-dependent mechanism, independent of insulin signaling and glucose metabolism. This effect is mediated by a transcriptional upregulation of ß secretase (BACE 1) which leads to an increase of ß amyloid [131]. However, when insulin is added to metformin, it potentiates insulin’s effects on amyloid reduction, improves neuronal insulin resistance, and impairs glucose uptake and AD-associated neuropathological characteristics by activating the insulin signaling pathway [129].

Metformin has been shown to promote rodent and human neurogenesis in culture by activating a protein kinase C-CREB binding protein (PKC-CBP) pathway, recruiting neural stem cells and enhancing neural function, particularly spatial memory function. It is noteworthy that neural stem cells can be recruited in an attempt of endogeneously repairing the injured or regenerating brain [132]. In the context of metformin’s potential neuroprotective effect *in vivo*, the capacity of the drug to cross the blood brain barrier needs to be further elucidated. Provided that this crossing could occur, metformin may become a therapeutic agent not only in peripheral and diabetes-associated vascular neuropathy but also in neurodegenerative diseases.

### Metformin and cancer

Patients with type 2 diabetes have increased risks of various types of cancer, particularly liver, pancreas, endometrium, colon, rectum, breast, and bladder cancer. Cancer mortality is also increased [133,134]. Many studies showed reduced incidence of different types of cancer in patients as well a reduced cancer-related mortality in patients using metformin (Table 3).

**Table 3:** Reduced incidence and cancer-related mortality in metformin treated patients

**Author**	**Study type**	**Tumor type**	**Region**	**Total participants**	**Follow up (years)**	Confounding adjustment *
Evans [135]	Pilot Observational Study	Not specified	Tayside, Scotland. UK	11,876	8	IMC, smoking, blood pressure, material deprivation
Bodmer [136]	Retrospective Case control	Breast	UK	22,661	10	Age, BMI, smoking, estrogen use, diabetes history, HbA1c, renal failure, congestive heart failure, ischemic heart disease
Li [137]	Prospective case–control	Pancreatic	USA	1,836	4	Sex, age, smoking, DM-2, duration of diabetes, HbA1c, insulin use, oral antidiabetic medication, IMC, risk factors
Donadon [138]	Retrospective Case–control	Hepatocellular carcinoma	Italy	1,573	12	Sex, age, BMI, alcohol abuse, HBV and HCV infection, DM-2, ALT level
Libby [139]	Retrospective cohort study	Colorectal	Scotland. UK	8,000	9	Sex, age, BMI, HbA1c, deprivation
						Other drug use

*Confounding adjustment: Adjustment of variables that could potentially interfere with cancer incidence.

The underlying mechanisms of tumorigenesis in T2DM seem to be related to insulin resistance, hyperinsulinemia, elevated levels of IGF-1 [140-142], and hyperglycemia with the latter driving ATP production in cancer cells through the glycolytic pathway, a mechanism known as the Warburg effect [142].

Metformin significantly reduces tumorigenesis and cancer cell growth although how it does it is not well understood. It may be due to its effects on insulin reduction and hyperinsulinemia, and consequently on IGF-1 levels, which have mitogenic actions enhancing cellular proliferation,but may also involve specific AMPK-mediated pathways [133].

Activation of AMPK leads to inhibition of mTOR through phosphorylaton and subsequent activation of the tumor suppressor tuberous sclerosis complex 2 (TSC2). The mTOR is a key integrator of growth factor and nutrient signals as well as a critical mediator of the PI3K/PKB/Akt pathway, one of the most frequently disregulated signaling pathways in human cancer [144]. Metformin may have additional anticancer properties independent of AMPK, liver kinase 1 (LKB1), and TSC2. This may be related, in part, to the inhibition of Rag GTPase-mediated activation of mTOR [145].

Patients with type 2 diabetes who are prescribed metformin had a lower risk of cancer compared to patients who did not take it. The reduced risk of cancer and cancer mortality observed in these studies has been consistently in the range of 25% to 30% [135-139,145-147]. An observational cohort study with type 2 diabetics who were new metformin users found a significant decrease in cancer incidence among metformin users (7.3%) compared to controls (11.6%). The unadjusted hazard ratio (95% CI) for cancer was 0.46 (0.40–0.53). The authors suggested a dose-related response [136]. In an observational study of women with type 2 diabetes, a decreased risk of breast cancer among metformin users was only seen with long-term use [137].

Metformin use is associated with lower cancer-related mortality. A prospective study (median follow-up time of 9.6 years) found that metformin use at baseline was associated with lower cancer-related mortality and that this association appeared to be dose dependent [138]. Diabetic patients with colorectal cancer who were treated with metformin had lower mortality than those not receiving metformin [139]. Patients with type 2 diabetes exposed to sulfonylureas and exogenous insulin had a significantly increased risk of cancer-related mortality compared with patients exposed to metformin. However, whether this increased risk is related to a deleterious effect of sulfonylurea and insulin or a protective effect of metformin or due to some unmeasured effect related to both choice of therapy and cancer risk is not known [147].

The proposed mechanisms of metformin anti-cancer properties are not fully understood. Most are mainly mediated through AMPK activation which requires LKB1, a well-known tumor suppressor [2]. Some of these mechanisms may be through inhibition of cell growth [148], IGF-1 signaling [149], inhibition of the mTOR pathway [150], reduction of human epidermal growth factor receptor type 2 (HER-2) expression (a major driver of proliferation in breast cancer) [151], inhibition of angiogenesis and inflammation [152], induction of apoptosis and protein 53 (p53) activation [153], cell cycle arrest [137,154], and enhancement of cluster of differenciation 8 (CD8) T cell memory [155].

### Future roles for metformin in cancer therapy

*In vitro* and *in vivo* studies strongly suggest that metformin may be a valuable adjuvant in cancer treatment. Some of the proposed future roles yet to be defined through further research are outlined as follows:

Tumor prevention

When compared to those on other treatments, metformin users had a lower risk of cancer. A dose-relationship has been reported [138,144,145].

Adjunct in chemotherapy

Type 2 diabetic patients receiving neo-adjuvant chemotherapy for breast cancer as well as metformin were more likely to have pathologic complete response (pCR) than patients not receiving it. However, despite the increase in pCR, metformin did not significantly improve the estimated 3-year relapse-free survival rate [156].

Tumor relapse prevention

Cancer stem cells may be resistant to chemotherapeutic drugs, therefore regenerating the various tumor cell types and promoting disease relapse. Low doses of metformin inhibited cellular transformation and selectively killed cancer stem cells in four genetically different types of breast cancer in a mouse xenograft model. The association of metformin and doxorubicin killed both cancer stem cells and non-stem cancer cells in culture. This may reduce tumor mass and prevent relapse more effectively than either drug used as monotherapy [157].

### Metformin contraindications

Metformin is contraindicated in patients with diabetic ketoacidosis or diabetic precoma, renal failure or renal dysfunction, and acute conditions which have the potential for altering renal function such as: dehydration, severe infection, shock or intravascular administration of iodinated contrast agents, acute or chronic disease which may cause tissue hypoxia (cardiac or respiratory failure, recent myocardial infarction or shock), hepatic insufficiency, and acute alcohol intoxication in the case of alcoholism and in lactating women [158]. Several reports in literature related an increased risk of lactic acidosis with biguanides, mostly phenformin, with an event rate of 40–64 per 100,000 patients years [159] whereas the reported incidence with metformin is 6.3 per 100,000 patients years [160].

Structural and pharmacokinetic differences in metformin such as poor adherence to the mitochondrial membrane, lack of interference with lactate turnover, unchanged excretion, and inhibition of electron transport and glucose oxidation may account for such differences [161].

Despite the use of metformin in cases where it is contraindicated, the incidence of lactic acidosis has not increased. Most patients with case reports relating metformin to lactic acidosis had at least one or more predisposing conditions for lactic acidosis [161].

Renal dysfunction is the most common risk factor associated with lactic acidosis but so far there is no clear evidence indicating at which level of renal dysfunction metformin should be discontinued or contraindicated in order to prevent lactic acidosis. Some authors have suggested discontinuing its use when serum creatinine is above 1.5 mg/dL in men and 1.4 mg/dL in women [103] while others suggested a cut-off of 2.2 mg/dL and continuous use even in the case of ischaemic cardiopathy, chronic obstructive pulmonary disease, or cardiac failure [84].

As serum creatinine can underestimate renal dysfunction, particularly in elderly patients and women, the use of estimated GFR (eGFR) has been advocated. The recommended eGFR thresholds are generally consistent with the National Institute for Health and Clinical Excellence guidelines in the U.K. and those endorsed by the Canadian Diabetes Association and the Australian Diabetes Society. Metformin may be continued or initiated with an eGFR of 60 mL/min per 1.73 m^2^ but renal function should be monitored closely (every 3–6 months). The dose of metformin should be reviewed and reduced (e.g. by 50% or to half-maximal dose) in those with an eGFR of 45 mL/min per 1.73 m^2^, and renal function should be monitored closely (every 3 months). Metformin should not be initiated in patients at this eGFR [162]. The drug should be stopped once eGFR falls to 30 mL/min per 1.73 m^2^. Frid *et al.* supports these recommendations through findings that above 30 ml/min/1.73 m^2^ metformin levels rarely goes above 20 mmol/l, which seems to be a safe level [163].

Another clinical condition associated with lactic acidosis in patients using metformin is heart failure [79].

### Adverse effects

Gastrointestinal intolerance occurs quite frequently in the form of abdominal pain, flatulence, and diarrhea [164]. Most of these effects are transient and subside once the dose is reduced or when administered with meals. However, as much as 5% of patients do not tolerate even the lowest dose [165].

About 10–30% of patients who are prescribed metformin have evidence of reduced vitamin B12 absorption due to calcium-dependent ileal membrane antagonism, an effect that can be reversed with supplemental calcium [166]. This vitamin B12 deficiency is rarely associated with megaloblastic anemia [167].

A multicentric study reported a mean decrease of 19% and 5% in vitamin B12 and folate concentration, respectively [168]. Vitamin B12 deficiency has been related with dose and duration of metformin use and occurs more frequently among patients that use it for more than 3 years and in higher doses [169].

Other adverse reactions are sporadic, such as leucocytoclastic vasculitis, allergic pneumonitis [170], cholestatic jaundice [171], and hemolytic anaemia [172].

Hypoglycemia is very uncommon with metformin monotherapy [173] but has been reported in combination regimens [174], likely due to metformin potentiating other therapeutic agents.

### Drug interactions

Clinically significant drug interactions involving metformin are rare. Some cationic agents such as amiloride, digoxin, morphine, procainamide, quinidine, quinine, ranitidine, triamterene, trimethoprim, and vancomycin that are eliminated by renal tubular secretion may compete with metformin for elimination. Concomitant administration of cimetidine, furosemide, or nifedipine may also increase the concentration of metformin. Patients receiving metformin in association with these agents should be monitored for potential toxicity. Metformin should be discontinued at least 48 hours prior to the administration of iodinated contrast media which can produce acute renal failure and should only be restarted if renal function is normal [175].

### Tolerability

Gastrointestinal side-effects are common with the use of metformin of standard release and are usually associated with rapid titration and high-dose initiation of metformin.

These effects are generally transient, arise early in the course of treatment, and tend to subside over time [176]. The gastrointestinal side-effects can be addressed by taking the agent with meals, reducing the rate of dose escalation, or transferring to a prolonged-release formulation [177].

Some studies point to a dose-related relationship of the incidence of side-effects [178] whereas other evidence gives no support for a dose-related effect of metformin on the gastrointestinal system [179].

### Metformin XR

The metformin XR formulation releases the active drug through hydrated polymers which expand after uptake of fluid, prolonging gastric residence time which leads to slower drug absorption in the upper gastrointestinal tract and allows once-daily administration [180].

A prospective open label study assessed metformin XR effectiveness on three cardiovascular risk factors: blood glucose (HbA1c, fasting blood glucose, and postprandial blood glucose); total cholesterol, LDL cholesterol, HDL cholesterol; and triglycerides and blood pressure. No significant differences were observed by any anthropometric, clinical, or laboratory measures except for plasma triglycerides which were lower in the group switched to metformin XR [181]. Metformin tolerability as well as patient acceptance was greater in the group switched to metformin XR. Other studies have found good to excellent glycemic control with metformin XR in type 2 diabetic patients who did not have well-controlled diet and exercise alone [182]. Metformin XR has been associated with improved tolerability [182] and increased compliance [183].

## Conclusions

In recent years, metformin has become the first-line therapy for patients with type 2 diabetes. Thus far, metformin is the only antidiabetic agent which has shown reduced macrovascular outcomes which is likely explained by its effects beyond glycemic control. It has also been employed as an adjunct to lifestyle modifications in pre-diabetes and insulin-resistant states. A large amount of evidence in literature supports its use even in cases where it would be contra-indicated mainly due to the fear of lactic acidosis which has been over-emphasized as the available data suggest that lactate levels and risk of lactic acidosis do not differ appreciably in patients taking this drug versus other glucose-lowering agents. It has also recently gained attention as a potential treatment for neurodegenerative diseases such as Alzheimer’s disease.

## Abbreviations

ACE: Angiotensin converting enzyme; AD: Alzheimeir disease; AGE: Advanced glycosilation end product; AMPK: Adenosine monophosfatase protein kinase; BACE 1: Beta-amyloid cleaving enzyme- 1; BMI: Body muscular index; BP: Blood pressure; CAD: Coronary artery disease; CDK: Cyclin dependent kinase; CHF: Cardiac heart failure; CPR: Complete pathologic response; CREB: cAMP responsive element binding protein; CsA: Cyclosporin A; CVD: Cardiovascular disease; DDPPIV: Dipeptidyl peptidase IV; DM: Diabetes mellitus; DYm: Mitochondrial membrane potential; FPG: Fasting plasma glucose; GDM: Gestacional diabetes mellitus; GFR: Glomerular filtration rate; eGFR: Estimated glomerular filtration rate; GLP-1: Glucagon like peptide 1; HAART: Highly active antiretroviral therapy; HALS: HIV associated lipodystrophy syndrome; HIV: Human imunodeficiency virus; HER-2: Human epidermal growth factor receptor type 2; HF: Heart failure; HOMA-IR: Homeostatic model assessment – insulin resistance; IFG: Impaired fasting glucose; IGT: Impaired glucose tolerance; LKB-1: Liver kinase 1; LSM: Lifestyle modification; MET: Metformin; MG: Metylglyoxal; mTOR: Mammalian target of rapamycin; NF-KB: Nuclear factor kappa beta; NRTIs: Nucleoside reverse transcriptase inhibitors; OGTT: Oral glucose tolerance test; PAI-1: Plasminogen activator inhibitor; PCOS: Policystic ovary syndrome; PIs: Protease inhibitors; PTP: Permeability transition pore; ROS: Reactive oxigen species; SIRT-1: Sirtuin 1; T2DM: Type 2 diabetes mellitus; TCS2: Tuberous sclerosis complex 2; TORC2: Transducer of regulated CREB protein 2; TZP: Triazepinone; UCPs: Uncoupled proteins; VEGF: Vascular endothelial growth factor; Vwf: Von Williebrand fator.

## Competing interests

The authors declare that they have no competing interests.

## Authors’ contributions

Lilian Beatiz Aguayo Rojas drafted the manuscript and Marilia Brito Gomes reviewed and edited the manuscript. Both authors read and approved the final manuscript.

## Authors’ information

Lilian Beatriz Aguayo Rojas is post graduate student at the State University of Rio de Janeiro, Diabetes Unit, Internal Medicine Department.

Marilia Brito Gomes is an Associate Professor at the State University of Rio de Janeiro, Diabetes Unit, Internal Medicine Department.
